# Directing spatial attention to locations within remembered and imagined mental representations

**DOI:** 10.3389/fnhum.2013.00154

**Published:** 2013-04-25

**Authors:** Simon G. Gosling, Duncan E. Astle

**Affiliations:** ^1^School of Medicine, University College CorkCork, Ireland; ^2^Department of Psychology, Royal Holloway, University of LondonEgham, Surrey, UK; ^3^MRC Cognition and Brain Sciences UnitCambridge, UK

**Keywords:** attention, ERPs, visual short-term memory, visual working memory, mental imagery

## Abstract

Spatial attention enables us to enhance the processing of items at target locations, at the expense of items presented at irrelevant locations. Many studies have explored the neural correlates of these spatial biases using event-related potentials (ERPs). More recently some studies have shown that these ERP correlates are also present when subjects search visual short-term memory (VSTM). This suggests firstly that this type of mental representation retains a spatial organization that is based upon that of the original percept, and secondly that these attentional biases are flexible and can act to modulate remembered as well as perceptual representations. We aimed to test whether it was necessary for subjects to have actually seen the memoranda at those spatial locations, or whether simply imagining the spatial layout was sufficient to elicit the spatial attention effects. On some trials subjects performed a “visual” search of an array held in VSTM, and upon other trials subjects imagined the items at those spatial locations. We found ERP markers of spatial attention in both the memory-search and the imagery-search conditions. However, there were differences between the conditions, the effect in the memory-search began earlier and included posterior electrode sites. By contrast the ERP effect in the imagery-search condition was apparent only over fronto-central electrode sites and emerged slightly later. Nonetheless, our data demonstrate that it is not necessary for subjects to have ever seen the items at spatial locations for neural markers of spatial attention to be elicited; searching an *imaginary* spatial layout also triggers spatially-specific attention effects in the ERP data.

## Introduction

Spatial attention enables us to bias incoming sensory information, in favor of that which is relevant to the task at hand (Posner, 1980). Recent research has shown that this is also possible for the content of mental representations that exist in the absence of concurrent visual input, namely in the case of visual short-term memory (VSTM; Griffin and Nobre, 2003; Matsukura et al., 2007; Makovski et al., 2008; Sligte et al., 2008, 2010; Astle et al., 2009a,b, 2010, 2012a,b; Kuo et al., 2009). These mental representations appear to retain something of the spatial layout of their original sensory antecedents (Harrison and Tong, 2009); similar spatial attention mechanisms can operate upon these VSTM representations as those that operate on sensory input (Kuo et al., 2009). In the current study we wanted to test whether or not that was the case for mental representations whose organization is not based directly upon sensory input, but in which the spatial organization is merely imagined.

There is a relatively large literature using event-related potential (ERP) measures to explore spatial attention mechanisms. In most of these studies subjects are presented with a display of target(s) amidst a set of distracters. Because of the contralateral organization of the human visual system, as subjects covertly search their sensory input for the target, waveforms recorded contra and ipsilateral to the spatial location of that target diverge. One such ERP effect is called the N2pc (Luck and Hillyard, 1994)—a relative negativity over the posterior scalp contralateral to the location of the stimulus, apparent between 180 and 300 ms following the onset of the search array (Jolicoeur et al., 2006, 2008; Eimer and Kiss, 2008, 2010a; Astle et al., 2009b). There are a number of other spatially-specific ERP components (i.e., that appear as a difference between contra and ipsilateral recordings, depending upon the spatial location of the target) that are evident in the early visual response, and which are thought to index the deployment of spatial attention processes. The early attention directing negativity (EDAN) is seen over posterior electrodes from 150 to 400 ms, much like the N2pc but cue-locked (Nobre et al., 2000; van Velzen and Eimer, 2003; Murray et al., 2011). Specifically the EDAN is thought to reflect voluntary spatial attention in the presence of a directional cue (Praamstra and Kourtis, 2010). Unlike some other supposed markers of spatial attention, the EDAN appears to be elicited only by spatial shifts of *visual* attention, indeed there is some controversy as to whether it reflects the visual characteristics of the cue itself rather than the shifting of spatial attention in anticipation of the target per se (van Velzen and Eimer, 2003). The anterior directing attention negativity (ADAN) occurs over fronto-central sites between 300 and 500 ms after cue onset, follows the EDAN, and is thought to reflect the voluntary initiation of attentional shifts within an anterior attention system in response to a cue (Nobre et al., 2000; Praamstra and Kourtis, 2010). Importantly, many researchers have observed the ADAN effect when subjects' attention is directed toward tactile or auditory stimuli, as well as upcoming visual stimuli (e.g., Eimer et al., 2002), leading to the suggestion that it reflects a supramodal attention control mechanism. Although this too has been questioned, with some arguing that this supposedly domain-general mechanism can be modulated by the modality of the expected stimuli and the response requirements of the subsequent task (e.g., Green et al., 2008). In summary, there are a number of different accounts as to what exactly these early ERP effects index, but for the purposes of this study their critical characteristic is that they are spatially specific. That is, these ERPs effects are all revealed by comparing recordings contra and ipsilateral to the actual physical location of the target within the visual display.

Recently it has been demonstrated that similar spatially-specific attention mechanisms are recruited when subjects search the spatial layout of VSTM (Astle et al., 2009b; Kuo et al., 2009). Participants were first presented with an array of to-be-remembered items. After a brief delay a single item (termed the probe) was presented at fixation. Participants were instructed to decide whether or not this probe had been one of the original memory items. Time-locking the ERP waveforms to the onset of this central probe, the authors compared recordings contra and ipsilateral to the item's *original* location within with the memory array. Surprisingly, this revealed an N2pc, with recordings being more negative contralateral to that item's original location (Astle et al., 2009b; Kuo et al., 2009). The implication is that subjects' VSTM of the original items retained a spatial layout similar to that of its original, such that when subjects performed the recognition judgment at probe onset they recruited spatial attention mechanisms. A number of additional studies have shown that later probe-locked components also reflect the spatial layout of the original memoranda. Eimer and Kiss (2010b) observed a sustained posterior contralateral negativity (SPCN), from around 300 to 600 ms following the onset of the centrally presented probe, which had a topography similar to that of the N2pc. They suggest that this reflects the *access* of contralateralized VSTM representations, thereby reflecting their spatiotopic organization. In short, much like there are spatially-specific ERP effects that occur when subjects bias sensory input (or prepare to do so in the case of the EDAN and ADAN), there are also spatially-specific ERP effects when participants attempt to access the content of VSTM.

It remains unclear whether mental imagery has the same spatial organization as perception, as VSTM is thought to. Here we attempted to address whether subjects “search” an imagined spatial layout of items, using those same spatially-specific attention markers as when accessing a remembered layout of items. One possibility is that the memory-search ERP effects described above do not depend upon subjects having actually seen the memoranda at those lateral locations, simply imagining them there may be enough to elicit the effect. Indeed some simple types of mental imagery and VSTM have much in common, with a number of studies providing evidence that both share a common neural code with perception (e.g., Harrison and Tong, 2009; Stokes et al., 2009).

We devised a paradigm in which participants were presented with items at the vertical midline. Arrows then indicated that these shapes were moving to lateral positions, and, in one condition (the “VSTM search” condition), the items appeared at these new lateral locations. However, in a second condition (the “imagery search” condition) the items never actually appeared at these lateral locations, but subjects were instructed to imagine them there. For both trial types the sequence concluded with a centrally presented probe shape and participants were instructed to make a judgment as to its location within their mental representation. In both conditions we time-locked our ERP waveforms to the onset of the final probe, and compared waveforms recorded contra or ipsilateral to that shape's previous remembered or imagined location. If the neural markers of spatial attention previously seen were not dependent on subjects actually having seen the items at lateralized locations, then we ought to see equivalent effects across these two conditions. A secondary question was whether there would be *any* contralateralized ERP effects in the imagery search condition at all (regardless of whether these were the same as those seen on the VSTM search trials), since this would indicate that when subjects make a judgment as to where they imagined that probe shape being, spatial attention (in some form) is recruited.

## Methods

### Participants

Thirty-one subjects completed an EEG recording session. Four subjects were removed due to excessive occulomotor artifacts, therefore data analyses were carried out on the data from 27 participants (15 female, mean age 20.41 ± 1.82 years, 25 right-handed and 2 left-handed). The study was approved by the ethical review panel at Royal Holloway, University of London. All subjects had normal or correct-to-normal vision and provided written informed consent. Each was paid £20 for participating in the study.

### Behavioral task

**Figure 1:**
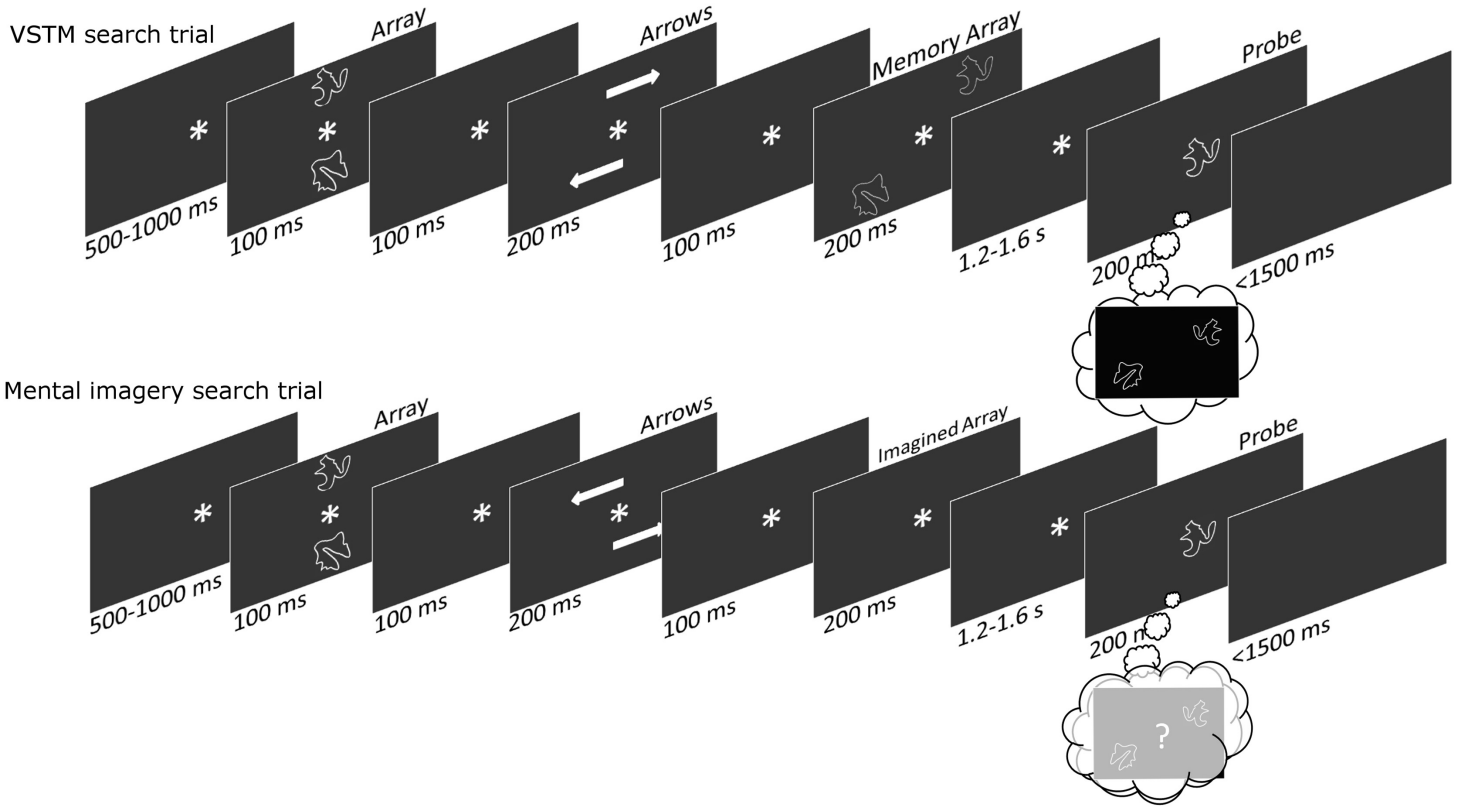
**A trial-order schematic showing the sequence of events within each trial.** On the VSTM-search trials subjects are presented with midline and then laterally located shapes, before being presented with a central probe shape (upon which they make a remembered location judgment). On the Imagery-search trials subjects are presented with midline but not laterally located shapes, before being presented with a central probe shape (upon which they make an imagined location judgment).

A trial order schematic can be seen in Figure 1. Participants were seated in dark room. On every trial subjects were presented briefly with two shapes at midline locations, above and below fixation. After a brief delay arrows appeared, indicating that the shapes were “moving” to new lateral locations. The arrows would either indicate that the top shape was moving to the top right and the bottom shape was moving to the bottom left location, or that the top shape was moving the top left and the bottom shape to the bottom right location. Subsequently, on half of the trials the shapes were actually faintly presented at these new lateral locations; on the other half of trials the shapes never actually appeared at these new lateral locations, but subjects were instructed to imagine them there nonetheless. After a “maintenance” delay, a single probe shape appeared at fixation and subjects had to respond as to this shape's location, either as they previously saw it or as they previously imagined it. We subsequently referred to these two tasks as the VSTM search and the mental imagery search, respectively; in the former subjects actually saw the shapes at lateral locations, whereas in the latter they only imagined them at these locations.

### Task design

All the timings for the trial sequences can be seen in the trial-order schematic in Figure 1. The shapes were drawn from a set of eight, with the two shapes being used on each trial always being different from one another (Endo et al., 2003). Each stimulus subtended a visual angle of 3.2° × 3° and was positioned at the corner of an invisible 8° × 8° square. The arrow stimuli subtended a visual angle of 4.1° × 1.3°, and the central asterisk was 1.4° × 1.3°. All stimuli were presented on a black background. There were an equal number of VSTM and imagery search trials, and an equal number of trials upon which the shapes “moved” to the top-right/bottom-left and top-left/bottom-right diagonals. Subjects responded using a rather complicated response-mapping. This was intentional, as we hoped that subjects might need to use a mental imagery strategy to help them select the correct response. When the shapes appeared (or were imagined) in the top-right/bottom-left diagonal subjects used the right hand, pressing the “M” key for probe items that appeared (or were imagined) in the right hemifield, and pressing the “N” key probe items that appeared (or were imagined) in the left hemifield; when the shapes appeared (or were imagined) in the top-left/bottom-right diagonal subjects used the left hand, pressing the “Z” key for probe items that appeared (or were imagined) in the left hemifield, and pressing the “X” key for probe items that appeared (or were imagined) in the right hemifield. Organizing the location-response mappings in this way meant that items from the left and right-hemifield were each responded to equally with both hands, ensuring that lateralized motor potentials did not interfere with the lateralized spatial attention effects. On one third of trials the probe shape was an item from the left-hemifield, on a third of trials the probe shape was an item from the right-hemifield, and on a third of trials the probe shape was neither of the original array items. On these probe-absent trials subjects ought to press nothing, and error rates on these trials were used to produce a false-alarm rate for our d' calculations (Green and Swets, 1966). Having some probe-absent trials also meant that subjects needed to remember/imagine the location of *both* array shapes; if there were no probe-absent trials then remembering/imagining the location of only one of the array shapes would be sufficient to perform the task. On each trial subjects were given feedback saying “Correct” or “Wrong.” We did this because the stimulus-response mapping was particularly difficult and we wanted subjects to keep performing at an optimal level. Subjects performed 400 trials, and took a self-paced break every 20 trials.

### EEG pre-processing and ERP formation

EEG data were acquired using a 64-channel Mark II Biosemi ActiveTwo system, at >2000 Hz. Additional electrodes were placed at the outer canthi of each eye and above and below the right eye, these were used to form HEOG and VEOG, respectively. Offline the data were down-sampled to 1000 Hz and re-referenced to the average of additional left and right mastoid electrode recordings. We applied a low-pass 40Hz filter and epoched the data from −200 to 500 ms relative to the onset of the probe. The recordings were baseline corrected from −200 to 0 ms. Artifacts were removed using an algorithm that detected peak to peak changes that exceed 130 μv; these were then checked manually to ensure that all trials contaminated by saccades and blinks were removed. We used a robust averaging technique that down-weighted the noisiest trials (those falling three standard deviations from the mean) within each condition. These averages were then combined using an averaging procedure that preserves the location of the electrode relative to the remembered/imagined location of the screen [contralateral or ipsilateral, as in Kuo et al. (2009)]. All of these steps were performed using SPM8 (http://www.fil.ion.ucl.ac.uk/spm/).

### ERP analyses

**Figure 2:**
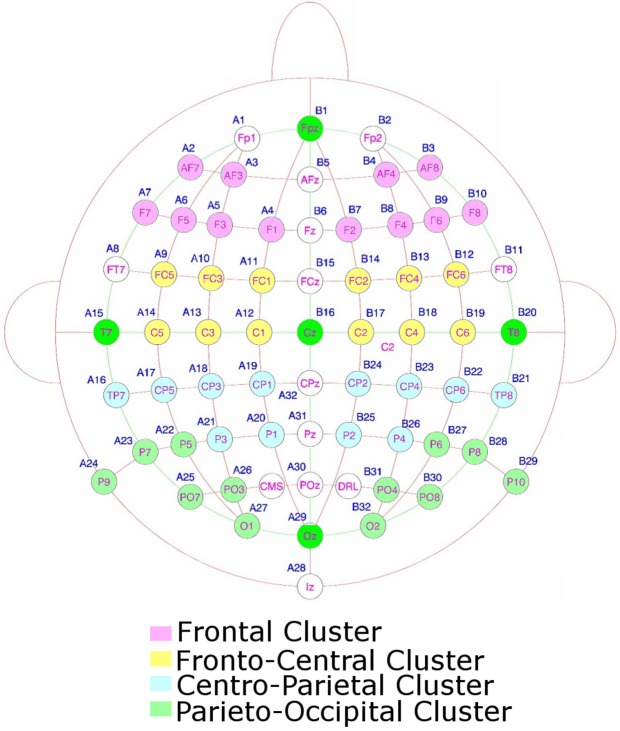
**The electrodes used to form the clusters for the statistical analysis**.

The recordings were grouped into four ipsilateral and four contralateral clusters of electrodes, each containing 6 electrodes (48 electrodes were used in the analysis in total). These clusters were as follows: a frontal cluster (comprising AF3/4, AF7/8, F1/2, F3/4, F5/6, F7/8); a fronto-central cluster (comprising FC1/2, FC3/4, FC5/6, C1/2, C3/4, C5/6); a centro-parietal cluster (comprising CP1/2, CP3/4, CP5/6, TP7/8, P1/2, P3/4); and a parieto-occipital cluster (comprising P5/6, P7/8, P8/9, PO3/4, PO7/8, O1/2). These clusters can be seen in Figure 2. We binned the data into 50 ms time bins. We submitted these binned averages to repeated measures ANOVAs for each time period from 200 ms onwards (Astle et al., 2009b; Kuo et al., 2009). In each condition (VSTM and imagery) we wanted to see whether there were any contra-ipsilateral differences as subjects searched the potentially spatially organized layout of the respective mental representation. So we submitted the voltages from each bin, in each condition, to a 2 (contra versus ipsilateral) × 4 (cluster) × 6 (electrode) ANOVA. (We included electrode as a separate factor within the ANOVA, although, to foreshadow the results, all of the significant effects were restricted to the cluster level). We also compared the two conditions directly by performing the same ANOVA with an additional two-level factor of VSTM versus Imagery search. Where appropriate the statistical tests were calculated using the Greenhouse–Geisser correction, to control for the potential non-sphericity of the data (Jennings and Wood, 1976). Because we had multiple time bins, it was important to correct for the number of independent comparisons that we made; a multi-step-up test was used to do this (Nichols and Hayasaka, 2003). The independent results were ranked by the significance of the effect, from the least to the most significant. In step one, the least significant result was tested for significance against a threshold (*p* = 0.05). If it proved to be non-significant, the next least significant *p*-value was selected (step 2) and multiplied by a scaling factor equal to the step number. This continues until one of the results survives the correction and all remaining (more significant) components were then classed as above threshold. A step-up correction was used as it is equivalent to the FDR corrections applied to traditional general linear model analyses (Nichols and Hayasaka, 2003).

## Results

### Behavioral results

**Figure 3:**
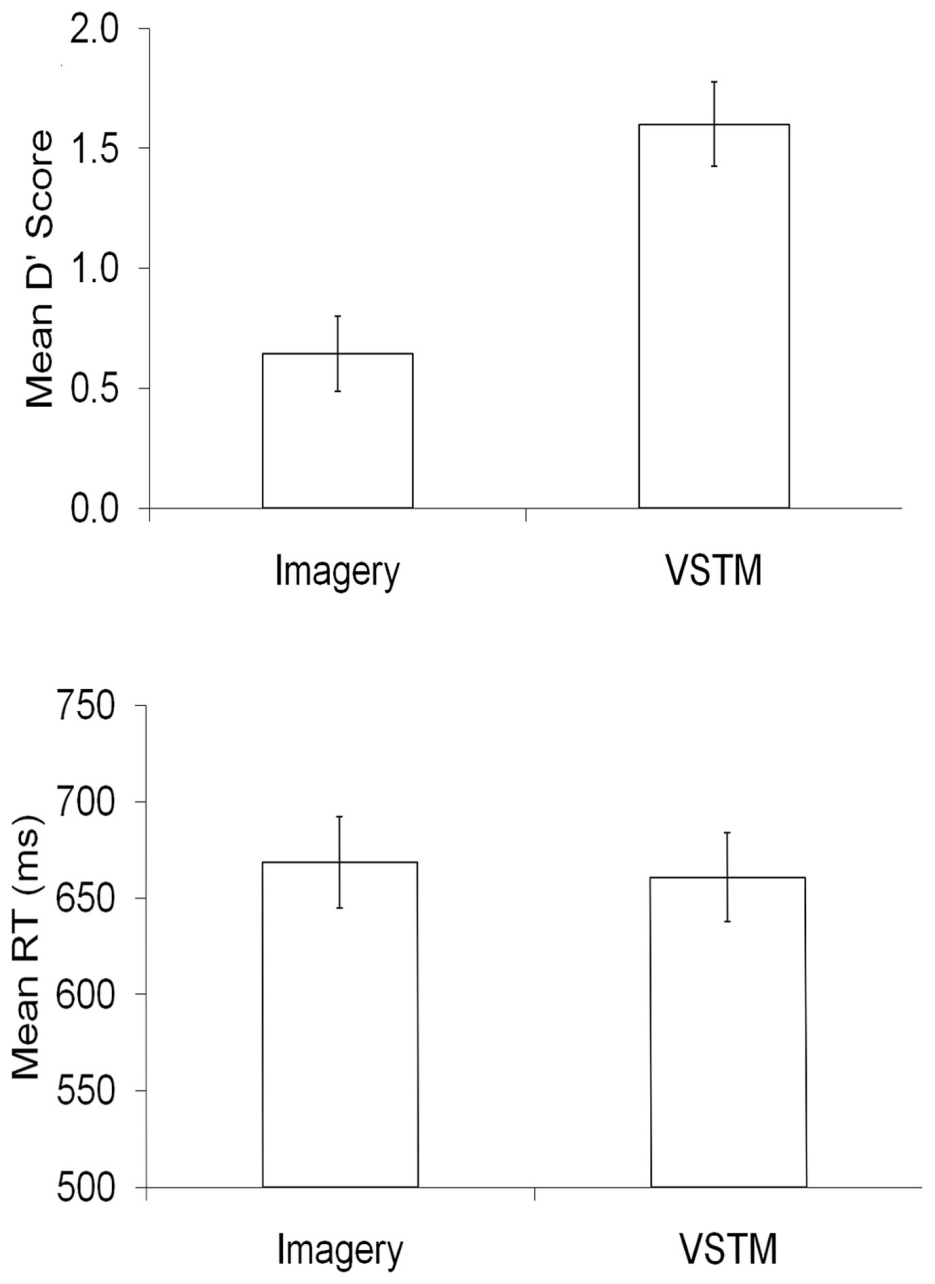
**The behavioral data, in terms of d prime and mean reaction times (RT), for each condition within the experimental task**.

We used error rates on the probe-absent trials as an index of false alarms, and accurate responses on probe-present trials as an index of correct hits. We used these to form d prime measures (Green and Swets, 1966). These scores were significantly higher on VSTM search trials, relative to Imagery search [*t*_(27)_ = 11.415, *p* < 0.001]. This was largely because Condition had a large effect on the false alarm rates (VSTM: 29%, Imagery: 50%), with there being less difference on the proportion of correct hits (VSTM: 85%, Imagery: 76%). There was no significant difference between correct trial RTs across the two conditions [*t*_(27)_ = 1.051, *p* = 0.303]. These data can be seen the Figure 3.

### ERP results

**Figure 4:**
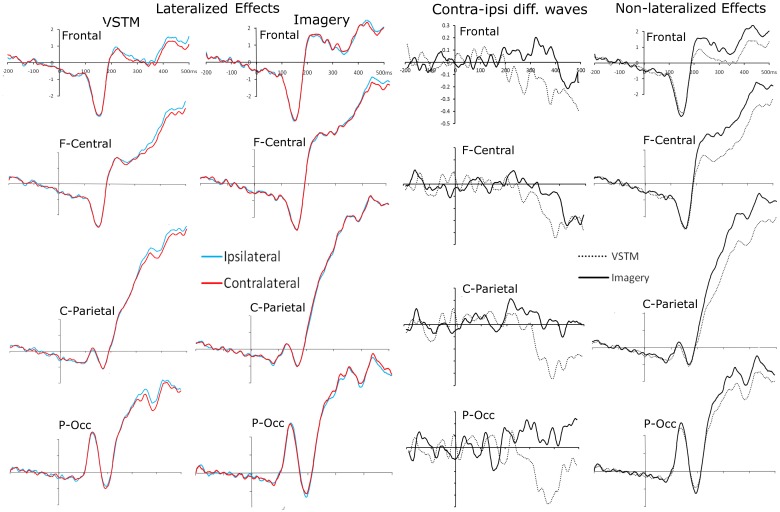
**Probe-locked waveforms for each of the four electrode clusters (Frontal, Fronto-Central, Centro-Parietal, Parieto-Occipital).** The left hand column shows the comparison of recordings taken ipsilateral and contralateral to the *remembered* location of the centrally presented probe shape. The middle-left column shows the comparison of recordings taken ipsilateral and contralateral to the *imagined* location of the centrally presented probe shape. The middle-right hand column shows the contralateral minus ipsilateral difference waves for each condition at each electrode cluster. The far right column shows a comparison of waveforms from the VSTM- and Imagery-search conditions, collapsed across the relative contralaterality of the recording.

We were primarily interested in whether ERPs recorded contra and ipsilateral to the probe shapes remembered or imagined location differed, as this would imply that spatial attention mechanisms were triggered when subjects searched these mental representations. Waveforms for each of the four clusters, comparing recordings contra and ipsilateral to the searched location can be seen in Figure 4. This figure also shows contralateral minus ipsilateral difference waves for each condition, as well as a non-lateralized comparison between the two search conditions (VSTM and Imagery search).

### Lateralized effects when searching VSTM and mental imagery

Whilst searching *remembered* lateral locations, from 350 ms until 500 ms subjects showed a significant effect of Contralaterality [*F*s > 6.934, *p*s_corrected_ < 0.05]. At no point did this interact with cluster [*F*s < 0.252, *p*s > 0.428]. Whilst searching *imagined* lateral locations, there was no significant main effect of Contralaterality in any bin, but between 400 and 450 ms the interaction between Contralaterality and cluster approached significance [*F*_(2.65, 68.98)_ = 2.486, *p* = 0.075] and it reached significance between 450 and 500 ms [*F*_(2.60, 68.01)_ = 4.703, *P*_corrected_ = 0.046]. In both cases this was because the fronto-central cluster showed a significant Contralaterality effect [*F*s > 4.406, *p*s < 0.046], whereas the other clusters did not [*F*s < 1.065, *p*s > 0.313].

### Direct comparison between VSTM and mental imagery search

In a subsequent analysis we included Condition (VSTM versus Imagery search) in the ANOVA. This revealed basic differences between these two trial types that did no interact significantly with Contralaterality. From 200 to 500 ms there was a significant main effect of Condition in every bin (VSTM versus Imagery) [*F*s > 10.730, *p*s < 0.003]. In all cases this was because the amplitudes for the Imagery trials were significantly greater than those for Memory trials (see Figure 4 far right panel). This analysis also revealed an interaction between Contralaterality and Condition; between 350 and 400 ms there was a significant interaction between Contralaterality and Condition [*F*_(1, 26)_ = 17.451, *P*_corrected_ = 0.006], which was driven by a simple main effect of Contralaterality in the VSTM search condition [*F*_(1, 26)_ = 14.196, *p* = 0.001], but not in the Imagery search condition [*F*_(1, 26)_ = 0.764, *p* = 0.390].

### Results summary

**Figure 5:**
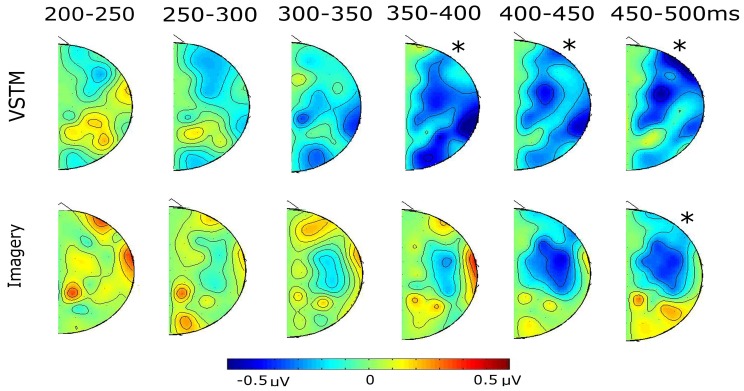
**The topographical distribution of the contra-ipsilateral differences (contra minus ipsi) for each condition, running from 200 ms onwards, in 50 ms bins.** In all cases the “^*^” indicates significant time-bins from the earlier statistical analysis.

Subjects were far more accurate at identifying the location of the probe within VSTM than within their Imagined mental representation. When subjects searched the layout of VSTM they showed contra-ipsilateral differences, with there being a negativity contralateral to the original lateral location of the probe shape, emerging from 350 until 500 ms, and was broadly distributed across the scalp. When subjects searched their imagined representation we also observed contra-ipsilateral differences, again, there was a negativity contralateral to the imagined lateral location of the probe shape, emerging from 400 until 500 ms. Unlike the VSTM search condition, the effect was significant at the fronto-central electrodes, and the imagery condition did not show the earlier contralaterality effect that we had seen in the VSTM condition. This can most clearly be seen in the difference waves shown in Figure 4. The topographies of the relative contra-ipsilateral difference across the consecutive time bins from 200 ms onwards can be seen in Figure 5.

## Discussion

Identifying the location of a target within the spatial layout of a VSTM representation is significantly easier than doing so when that spatial layout is only imagined. When subjects searched their VSTM representation we observed an SPCN-like contralateralized ERP effect, emerging at around 350 ms. The ~100 ms delay relative to previous VSTM-search studies (relative to Astle et al., 2009b; Kuo et al., 2009) perhaps stems of a difference of task (here subjects had to identify the probe's location, rather than just its presence in the VSTM array) or from the differences in stimuli (here the VSTM items were dimmed when presented). Alternatively it could be that this effect indexes a subtly different and later process. This posterior effect was accompanied by an ADAN-like negativity over the fronto-central electrodes. It is not clear whether these effects index target-selection, distracter suppression, or processes that lead up to these outcomes. The issue of what exact mechanisms these ERP effects index aside, their presence demonstrates clearly that when searching VSTM spatial attention mechanisms are recruited. A view growing in popularity is that VSTM is underpinned by the domain-specific sensory areas that originally processed the memoranda interacting with domain-general control areas, such as posterior parietal cortex and prefrontal cortex (Gazzaley and Nobre, 2011). Our result, demonstrating that spatially-specific attentional biases can operate on this VSTM representation, is consistent with this view (see also Astle et al., 2009b; Kuo et al., 2009).

There were clear differences between the ERP effects elicited by the probe, depending on whether subjects were accessing their memory of the spatial layout, or were simply imagining the spatial layout. The effect emerged significantly earlier in the VSTM-search than in the Imagery-search condition. The distribution of the effects also appeared to be different, with the effect in the Imagery-search condition being much more like a typical ADAN (Seiss et al., 2009), whereas the VSTM-search effect included an SPCN-like posterior effect.

A number of researchers have argued that visual mental imagery recruits those same early sensory processing mechanisms as are recruited when we perceive real stimuli (Kosslyn et al., 1993). In recent years there have been a number of demonstrations, using multi-voxel pattern analyses, that VSTM representations use the same sensory coding patterns as their perceptual antecedents (Harrison and Tong, 2009; Serences et al., 2009). Similar demonstrations have been made in the case of mental imagery (Stokes et al., 2009). For this reason one might suppose that participants are capable of using attentional mechanisms to bias these mental representations, in much the same way that they can bias sensory processing. However, in our data this appears not to be the case. When searching VSTM we observed an SPCN-like ERP effect that included the electrodes across the posterior scalp sites. Whereas in the imagery search condition we only observed a more anterior ADAN-like effect. Previous work as shown that the SPCN is present when subjects access the spatiotopically organized content of VSTM (Eimer and Kiss, 2010b). Here, we show that actually having seen the items at those lateral locations is necessary for this effect—merely imagining them there is not sufficient.

The ADAN is typically seen following a directional cue, as subjects prepare to select targets at a particular spatial location (Nobre et al., 2000). Other studies have shown that the ADAN is sensitive to the filtering demands of the task, as subjects attempt to select task-relevant items amidst distracters (Seiss et al., 2009). One view of the ADAN is that it reflects a supramodal attention control mechanism, because similar lateralized effects are seen regardless of the modality (Seiss et al., 2007). Conversely, some have argued that the effect is likely produced by multiple generators, which will reflect the changing response requirements and modality that are specific to each task (e.g., Green et al., 2008). In short, the effect likely reflects more than one process (Nobre et al., 2000). Indeed, even within this study we cannot definitely say whether or not the anterior effect seen in both the VSTM and Imagery-search conditions reflects the same process in each case. Nonetheless, our data demonstrate that an actual spatial layout is not necessary to achieve an ADAN-like effect, which we think will add to the on-going debate as to what exactly this anterior marker indexes. Indeed in the Imagery-search condition participants had only seen those items presented at the midline locations (which could therefore not elicit lateralized ERP effects)—the only spatial information present on these trials resulted from subjects' imagined mental representation. (Certainly spatial information was also present in the form of the arrows, but this was not specific to the probe item.) Of course the effect could be purely epiphenomenal; subjects may perform some non-spatial computation to select the response that they ought to make, and then shift their attention to the corresponding location on the screen. Whilst it is exceptionally difficult to demonstrate that the shifts in attention were to imagined objects at spatial locations per se, we think that this alternative explanation is unlikely. Firstly because our subjects reported imagining the spatial layout, and secondly because their responses were no slower in the imagery-search condition than they were in the VSTM-search condition—were they performing an extra non-spatial computation first, and only shifting their attention subsequent to this, then we would expect them to be slower in responding.

## Conclusion

In line with previous studies we show here that covertly searching for an item in VSTM elicits ERP markers of spatial attention. Furthermore, we demonstrate that ERP markers of spatial attention can be elicited even when subjects had never actually seen the items at lateralized spatial positions, simply imagining them there is sufficient. These data extend previous demonstrations that attentional biases are particularly flexible, demonstrating that they can be applied to either remembered or imagined mental representations.

### Conflict of interest statement

The authors declare that the research was conducted in the absence of any commercial or financial relationships that could be construed as a potential conflict of interest.
